# Editorial: Thermophilic and Halophilic Extremophiles in Eurasian Environments

**DOI:** 10.3389/fmicb.2019.00379

**Published:** 2019-03-05

**Authors:** Hongchen Jiang, Wen-jun Li, Nils-Kåre Birkeland, Dilfuza Egamberdieva

**Affiliations:** ^1^State Key Laboratory of Biogeology and Environmental Geology, China University of Geosciences, Wuhan, China; ^2^State Key Laboratory of Biocontrol and Guangdong Provincial Key Laboratory of Plant Resources, School of Life Sciences, Sun Yat-Sen University, Guangzhou, China; ^3^Southern Laboratory of Ocean Science and Engineering, Zhuhai, China; ^4^Department of Biological Sciences, University of Bergen, Bergen, Norway; ^5^Leibniz Centre for Agricultural Landscape Research, Müncheberg, Germany; ^6^Faculty of Biology, National University of Uzbekistan, Tashkent, Uzbekistan

**Keywords:** saline/hypersaline environments, thermal environments, microbial diversity, adaptation, pathways

Saline/hypersaline and thermal ecosystems are typical extreme environments. Microbial diversity, ecological function and adaptation in (hyper)saline and thermal ecosystems are attracting more attentions due to the following reasons: (1) (hyper)saline and geothermal ecosystems are analogs to certain extreme environments on early Earth and/or other planets and are thus suitable environments for analog studies on life origin and evolution and extraterrestrial life exploration; (2) microbial community complexity is relatively low in (hyper)saline and thermal ecosystems and thus they are often treated as model ecosystems for environmental microbiologists and biogeochemists to study how microbially mediated element cycling and biogeochemical processes respond to environmental variables (e.g., salinity, temperature); and (3) (hyper)saline and thermal ecosystems are inhabited by abundant and diverse microbial resources that have extensive biotechnological and commercial values.

The Eurasian continent possesses geologically and physiochemically diverse and unique terrestrial saline/hypersaline and thermal environments, which in general have been investigated for microbiology and biogeochemistry to a less extent than those in America. However, research results from Eurasian (hyper)saline and thermal ecosystems are essential to the field. Recently, a series of significant advances have been made on microbial diversity, ecological functions, and biogeochemistry in Eurasian (hyper)saline and thermal ecosystems with the use of next generation sequencing, omics technologies, and interdisciplinary collaboration.

We proposed this Research Topic to highlight the current advances and knowledge related to thermophilic and halophilic extremophiles in Eurasian environments. In this Research Topic “Thermophilic and Halophilic Extremophiles in Eurasian Environments” we accepted 11 original research articles that focus on microbial diversity and ecology, microbe-environment interactions, adaptation and evolution, element cycling, and potential biotechnological and industrial applications of thermophiles and halophiles from Eurasian environments. We are grateful to all authors who have contributed to this Research Topic. We are also grateful to all reviewers and editorial staff who have contributed during the reviewing and article production processes.

In addition, this Research Topic is one of the special issues set up at the fifth and sixth annual meetings of the Chinese Society of Geomicrobiology (CSG), which is an academic exchange platform for Chinese and international geomicrobiological scientists of related disciplines including mineral petrology, mineral deposits, paleontology, organic geochemistry, molecular paleontology, biogeochemistry, petroleum geology, microbiology, and ecology. This Research Topic is also one of the designated targets of the project “Network for improving research-based higher education in basic and applied microbiology,” funded by the Norwegian Eurasia program, using extremophiles as model microorganisms.

Among the accepted articles in this Research Topic, four and seven of them are about halophiles/microbial studies in (hyper)saline ecosystems and thermophiles/microbial studies in thermal ecosystems, respectively. Among the articles related to halophiles/microbial studies in (hyper)saline ecosystems, Liu et al. disclose the osmo-adaptive mechanisms of halophilic endophytic fungi from the molecular perspective. Yang et al. characterized Microbial benthic algal community structures in saline and hypersaline lakes and assessed how they were influenced by spatial and environmental factors on the Qing-Tibetan Plateau. Liu et al. reported that macro- and micro-elements predominate in shaping prokaryotic and fungal diversity and community composition in hypersaline sediments and saline-alkaline soils. Al-Mailem et al. found that addition of ferric sulfate and proline could assist halophilic/halotolerant soil microorganisms to tolerate high concentration of heavy metals, which could be potentially employed in bioremediation of spilled oil.

Among the articles related to thermophiles/microbial studies in thermal ecosystems, Zhang et al. reported on the diversity and composition of microbial communities in Tibetan hot springs and disclosed their different response patterns to environmental and spatial factor from the perspective of abundant and rare biospheres. Chan et al. reported on the prokaryotic biodiversity and its influencing factors in Malaysian hot springs. Wu et al. reported on the role of one thermophilic sulfate-reducing bacterium in the formation of thioarsenates, major As-S-containing compounds ubiquitously distributed in hot spring ecosystems globally. Toshchakov et al. reconstructed the energy metabolism of *Carboxydocella thermautotrophic* that coupled hydrogenogenic CO oxidation with the reduction of Fe(III) minerals from the genomic perspective. Gavrilov et al. presented the multi-omics analysis results on the respiratory pathways of a metabolically versatile thermophilic bacterium isolated from a deep thermal aquifer. Elcheninov et al. reported on the degradation pathways of Xanthan gum by a thermophilic bacterium retrieved from a hot spring in the Far-East of Russia with the use of comparative genomic and transcriptomic techniques. Chades et al. presented the ability of thermophilic anaerobic *Clostridia* to produce bioethanol by fermenting mannitol and mannitol-containing algal extracts.

We are delighted to present this Research Topic in Frontiers in Microbiology. We hope that this Research Topic will be interesting and useful to the readers of the journal and broaden the knowledge of thermophilic and halophilic extremophiles in Eurasian Environments. The findings presented in this Research Topic are promising but still limited. In the future, application of innovative research techniques and intensive and deep international collaborations will undoubtedly unveil more exciting aspects of thermophilic and halophilic extremophiles in Eurasian environments.

## Author Contributions

HJ organized this topic and wrote the editorial article. WL, N-KB, and DE are co-editors of the topic and discussed the writing.

### Conflict of Interest Statement

The authors declare that the research was conducted in the absence of any commercial or financial relationships that could be construed as a potential conflict of interest.

